# Analysis of metabolic differences in Tibetan medicinal plant *Phlomoides rotata* leaves in different habitats based on non-targeted metabolomics

**DOI:** 10.3389/fpls.2025.1503218

**Published:** 2025-04-08

**Authors:** Lele Wang, Hongli Wang, Junlin Chen, Yuzhen Lamu, Xiangyang Qi, Lei Lei, Kangshan Mao, Sonam Tso

**Affiliations:** ^1^ Key Laboratory of Biodiversity and Environment on the Qinghai-Tibetan Plateau, Ministry of Education, School of Ecology and Environment, Xizang University, Lhasa, China; ^2^ Lhasa, Urban Wetland Ecosystem, Observation and Research Station of Tibet Autonomous Region, Lhasa, China; ^3^ Fruit and Vegetable Breeding Laboratory, Qinzhou Branch of Guangxi Academy of Agricultural Sciences/Qinzhou Institute of Agricultural Sciences, Qinzhou, China; ^4^ Key Laboratory of Bio-Resource and Eco-Environment of Ministry of Education, Sichuan Zoige Alpine Wetland Ecosystem National Observation and Research Station, College of Life Sciences, Sichuan University, Chengdu, China

**Keywords:** Tibetan medicine, *Phlomoides rotata*, metabolomics, altitude gradient, slope direction, differential metabolites

## Abstract

*Phlomoides rotata*, a traditional Tibetan medicinal herb renowned for its anti-inflammatory and analgesic properties, exhibits distinct metabolite profiles across heterogeneous environments. However, the impacts of altitude and slope orientation on its secondary metabolism remain poorly understood. This study aimed to characterize metabolite variations in the leaves of *Phlomoides rotata* under different elevations and microclimates, providing a mechanistic basis for its quality evaluation and sustainable utilization. Metabolomic analysis was conducted using ultra-high-performance liquid chromatography-quadrupole time-of-flight mass spectrometry (LC-MS). Leaf samples were collected from three altitude gradients (4,300 m, 4,600 m, 5,000 m) and two slope orientations (south vs. north) in Budanla Mountain, Qusong County, Shannan, Xizang Autonomous Region, China. A total of 2,331 metabolites were detected, with lipids (41.93%), organic oxygen compounds (13.95%), and phenylpropanoids (12.4%) dominating the profile. Altitudinal gradients induced significant changes in 5 differentially accumulated metabolites (DAMs), including procyanidin B2 and dihydrocoumarin. Slope orientation influenced 17 DAMs, such as 2,3-secoporrigenin and 2-O-α-D-galactopyranosyl-1-deoxynojirimycin. Kyoto Encyclopedia of Genes and Genomes (KEGG) enrichment analysis revealed altitude-specific enrichment in flavonoid biosynthesis and pantothenate/CoA biosynthesis, while slope-related DAMs were enriched in glycerophospholipid metabolism and galactose metabolism. Altitude-driven increases in flavonoids (e.g., procyanidin B2) likely reflect adaptive responses to UV radiation and oxidative stress. Slope-related metabolite shifts, particularly glycerophospholipids, may relate to microclimate differences in temperature and moisture. These findings highlight the critical role of environmental factors in shaping the metabolic phenotype of *Phlomoides rotata*, with implications for pharmacologically active compound biosynthesis. The identified DAMs serve as potential biomarkers for quality control, while pathway analysis provides targets for metabolic engineering in conservation and cultivation practices.

## 1 Introduction

Tibetan medicinal plants are mainly distributed in the alpine grasslands, forests, farmlands, wetlands, and rivers of the Himalayas, Karakoram Range, Pamir Plateau, Altun Mountains, Qilian Mountains, Heng Duan Mountains, Kunlun Mountains, Tanggula Mountains, and Bayan Kala Mountains, which are located in higher altitudes. In recent years, scientists have been constantly checking and checking the plant resources of the Qinghai–Tibet Plateau and found that there are approximately 2,085 species of Tibetan medicine plants in 191 families and 692 genera distributed in the Qinghai–Tibet Plateau (Shang et al., 2006).

Tibetan medicine plant *Phlomoides rotata* is a perennial herb of Fabaceae Phlomoides, which is a traditional Tibetan medicine (Editorial Board of Flora of China, 1997). It often grows in the elevation of 3,900–5,100 m of stone alpine meadow, beach, or intensity-weathered gravel beach, where the harsh climate, extreme environment, low temperature, strong wind, high altitude, and other factors together shape this unique ecological environment. It is under these harsh conditions that *Phlomoides rotata* stubbornly survives and evolves, containing special mechanisms for adapting to extreme environments and accumulating unique chemical compositions and medicinal activities. *Phlomoides rotata* has been proved to have hemostatic, anti-inflammatory, analgesic, lipid-lowering, and immune-enhancing effects and has significant medicinal and economic value. At present, Researchers’ research on the medicinal plant *Phlomoides rotata* mainly focuses on resource distribution, chemical composition, clinical applications, and genetic diversity (Zheng et al., 2021). Ding Rong combined machine vision recognition with UAV remote sensing technology to identify *Phlomoides rotata* in plant communities through the artificial neural network algorithm and established Phlomoides in remote sensing areas. The method of rapid identification and calculation of plant number, leaf area, yield, and population spatial distribution pattern of rotata plants has realized the rapid and accurate estimation of wild resource reserves and population spatial distribution pattern of *Phlomoides rotata* (Ding, 2021). Li Maoxing et al. for the first time used liquid mass spectrometry (HPLC-MS) to rapidly analyze the iridoid glycosides in *Phlomoides rotata* and its preparations and isolated and identified five major iridoid glycosides. It was confirmed that the water extract of *Phlomoides rotata* and total iridoid glycosides had a good hemostatic effect. Total iridoid glycosides were the active site of *Phlomoides rotata*, whereas total flavonoids and large polar parts had no obvious hemostatic effect. It was found that the content of the active components of *Phlomoides rotata* was significantly higher in the aboveground part of *Phlomoides rotata* than in the root (Li, 2008). Li Zhijun et al. used various chromatographic techniques to isolate and purify the aboveground parts of *Phlomoides rotata* and identified the structure of the compounds according to physicochemical properties and spectral analysis. A total of 12 compounds were isolated from the ethanol extract of *Phlomoides rotata*, and the NO-releasing activity of mouse macrophage RAW264.7 was tested. It was found that the anti-inflammatory mechanism of *Phlomoides rotata* may be related to the inhibition of NO biosynthesis, and the active components of this mechanism are mainly flavonoids rather than iridoid components (Li et al., 2008). Li Tong conducted basic research on the anti-rheumatoid arthritis effects and substances of *Phlomoides rotata* extract, and the results showed that the *Phlomoides rotata* extract can significantly improve rheumatoid arthritis and has potential therapeutic effects on rheumatoid arthritis in both male and female pathological models. The active components and targets of the *Phlomoides rotata* extract against rheumatoid arthritis were further identified through network pharmacology and molecular docking. The results suggest that *Phlomoides rotata* in the treatment of rheumatoid arthritis may act on steroid-related pathways, and its active components are mainly flavonoids (Li, 2022). Wang Jing used a simple sequence repeat interval amplification (ISSR) molecular marker to analyze the genetic diversity of 10 different *Phlomoides rotata* populations in Yushu, and the study showed that the genetic distance of 10 *Phlomoides rotata* populations in Yushu was between 0.0076 and 0.2061. The average genetic distance between the populations was 0.1052, and the polymorphic site rate (PPL) was as high as 99.47%. The 10 *Phlomoides rotata* populations had high genetic diversity (Wang, 2014). In the 2010 edition of the Pharmacopoeia of the People's Republic of China, the specified source of *Phlomoides rotata* was modified from "the whole herb" to "the aboveground part". As a result, conducting in-depth research on the aboveground parts of *Phlomoides rotata*, particularly its leaves, holds great significance (Commission, 2010).

Metabolomics is a discipline to find the relative relationship of metabolites and physio-pathological changes through quantitative and qualitative analyses of all metabolites in an organism (Fiehn et al., 2000). At present, many medicinal, food, and industrial raw materials are the metabolites of plants, and the metabolic substances play an important role in human daily life. Metabolic substances are the active components of ethnic medicinal plants, which can not only be used in clinical practice and identify species types but also play an irreplaceable role in assisting plants to adapt to the complex environment. Untargeted Metabolomics refers to the research method that detects and analyzes all small-molecule metabolites in the sample without target and excavates metabolic profile differences by comparing between groups (Zhang et al., 2024). Bentley et al. used non-targeted liquid chromatography-tandem-mass spectrometry (LC-MS/MS) techniques to analyze Myrothamnus, a desiccation-tolerant medicinal shrub collected from three different climatic regions. A total of 41 suspected phenolic compounds were detected in flabellifolia materials, mainly flavonoids, nine of which were anthocyanins. The significant differences of M. flabellifolia phenols in different regions were revealed (Bentley et al., 2019). Stierlin et al. used automated thermal desorption-gas chromatography-mass spectrometry (ATD-GC-MS) technology to analyze lavender by dynamic headspace extraction. Field extraction and analysis of volatiles from lavandin showed that metabolomics techniques could effectively identify chemical differences between infected and healthy plants in complex field environments (Stierlin et al., 2020). Metabolomics methods have also been used to study the nutritional potential of medicinal plants from different environments, so as to identify differential metabolites. For example, Rashid et al. conducted non-targeted metabolomics studies on two types of hemp seeds from different environments based on GC-MS, and a total of 236 metabolites were detected. There were significant differences among 43 metabolites (P ≤ 0.05). Through the qualitative and quantitative accumulation of amino acids, cannabinoids, alkaloids, and fatty acids with important nutritional value, it was found that the high-altitude temperate Himalayan variety (CAN2) was superior to the low-altitude subtropical variety (CAN1), which confirmed that the environment had a significant influence on the antioxidant and nutritional value of hemp seeds (Rashid et al., 2021).

Metabolomics analysis can also study the chemical composition of various medicinal plants, providing insights for authenticity identification of plant samples and identification of bioactive compounds. Duarte et al. in selected ion monitoring (SIM) mode, two species, Maytenus ilicifolia and Maytenus aquifolium, were identified and quality-controlled using ultra-high-performance liquid chromatogen-mass spectrometry (UHPLC-MS), whereas extracts were analyzed in full-scan mode. To establish and test an analytical method that could quantify the content of catechin and epicatechin in dry Maytenus spp. leaves and simultaneously obtain their chemical profile to determine the authenticity of the leaf samples by using untargeted metabolomics, it was observed that the chemical profile of most of the samples was not compatible with M. ilicifolia leaves, indicating the need for stricter quality control of this material (Duarte et al., 2022). Doan et al. analyzed metabolites of Allium hookeri and identified new compounds in plants by using the molecular network of HRESI-qTOF MS/MS using non-targeted metabolomics methods, and isolated 10 compounds. Including one novel flavonoid (2) and nine known compounds (1 and 3–10), the phenolamides in Allium hookeri were found to have the potential as bioactive compounds to mitigate aging-related diseases (Doan et al., 2023). At the level of Tibetan medicine, it is believed that there is a difference in the efficacy between artificially cultivated *Phlomoides rotata* and wild *Phlomoides rotata*. In this paper, we used LC-MS technology to investigate whether there is a difference in the untargeted metabolites in the leaves of wild *Phlomoides rotata* at different altitudes and different slope orientations, as well as the compositions of the differential metabolites and the related pathways. This is intended to provide theoretical basis for selecting *Phlomoides rotata* with optimal pharmacological effects.

## 2 Materials and methods

### 2.1 Experimental material

**Figure 1:**
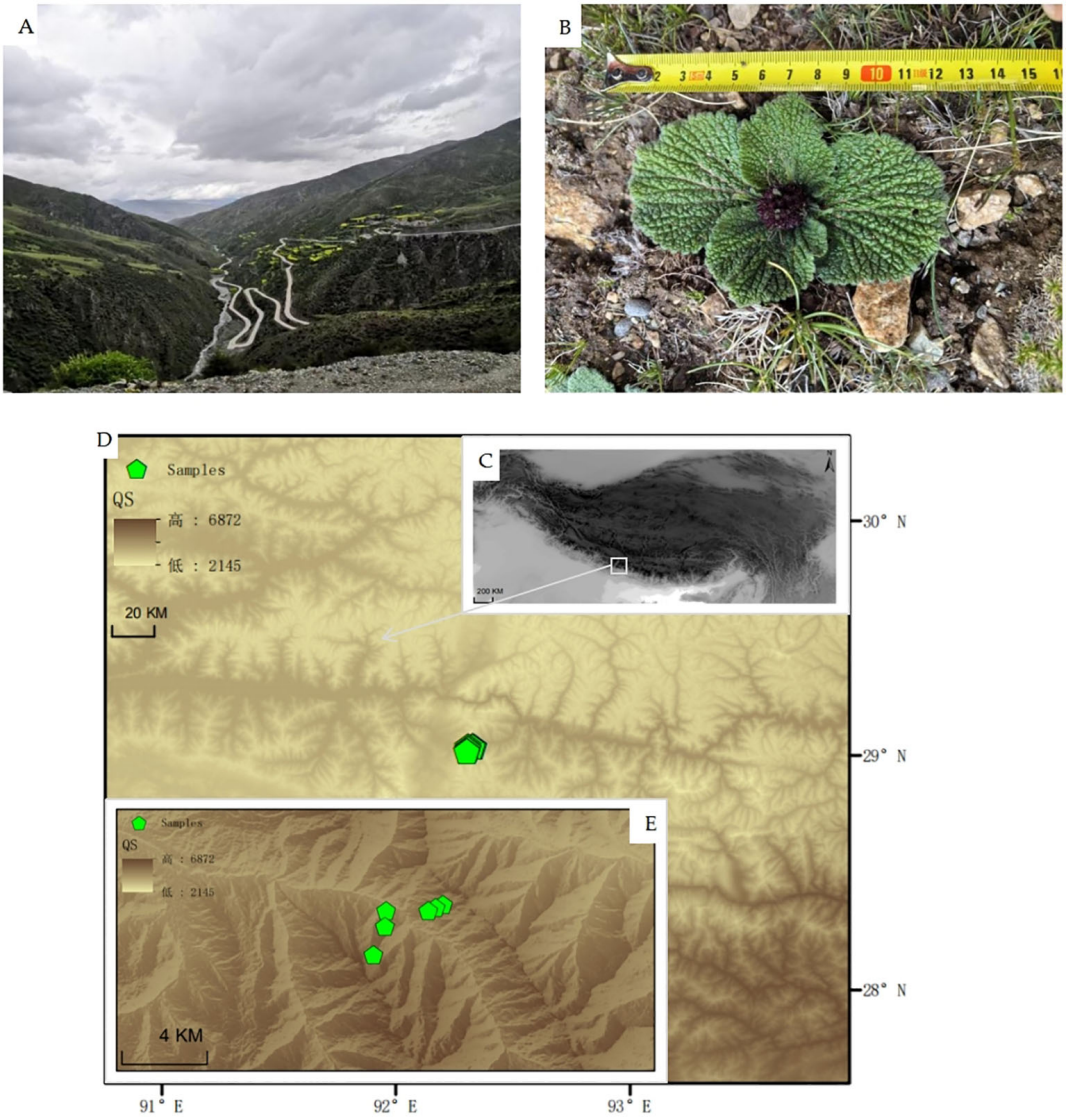
Elevation map of the sampling site. **(A)** Field-shot pictures of Budanla Mountain; **(B)** *Phlomoides rotata*; **(C)** Elevation map of the Qinghai–Tibet Plateau; **(D)** Elevation map of Qu song County; **(E)** Elevation map of Budanla Mountain and distribution of sampling sites (green icons indicate sampling points).

Sampling was conducted in September, when the aboveground biomass of alpine plants was highest, starting from 5 September 2022. The sampling site was located in Budanla Mountain, Qu song County, Shannan City, Xizang, China, on the eastern edge of the Himalayas. The natural habitats and elevation map of the sampling sites are shown in **Figure 1**. On both the north and south slopes at elevations of 4,300–4,600–5,000 m, six plots each with an area of 100 m × 20 m were established at intervals of 300 m, *Phlomoides rotata* aboveground parts were collected. The longitude, latitude, altitude, and main associated vegetation of each plot were recorded. The associated vegetation in the plot at an altitude of 5,000 m mainly comprised *Saussurea leontodontoides* (DC.) Sch. Bip., *Leontopodium nanum* (Hook. f. & Thomson ex C. B. Clarke) Hand.-Mazz., *Bistorta vivipara* (L.) Gray, etc. The associated vegetation in the plot at an altitude of 4,600 m mainly consisted of *Potentilla saundersiana* Royle, *Carex myosuroides* Vill., and *Cyananthus incanus* Hook. f. & Thomson, *Tibetia himalaica* (Baker) H. P. Tsui, etc. The associated vegetation in the plot at an altitude of 4,300 m mainly included *Juniperus procumbens* (Siebold ex Endl.) Miq., *Spiraea salicifolia* L., *Bistorta vivipara* (L.) Gray, and *Trachydium subnudum* C. B. Clarke ex H. Wolff. Within each plot, five leaves of *Phlomoides rotata* plants with similar population density, growth vigor, and growth stage were randomly selected at intervals of 20 m, resulting in a total of 30 leaf samples. These samples were brought back using an on-board refrigerator for subsequent testing. The sampling information is shown in **Table 1**.

**Table 1:** Statistics of sampling information.

Place	Aspect	Altitude/m	Longitude	Latitude
Qu song County Budanla mountain	S	4,300	E92°20′01. 91″	N29°2′19. 62″
		4,600	E92°19′50. 90″	N29°2′15. 03″
		5,000	E92°19′39. 89″	N29°2′10. 44″
	N	4,300	E92°18′35. 73″	N29°2′10. 35″
		4,600	E92°18′33. 92″	N29°1′45. 58″
		5,000	E92°18′16. 39″	N29°1′02. 40″

### 2.2 Instruments and equipment

The instruments used for this study were a New Classic MF MS105DU Electronic balance (METTLER TOLEDO, China), a Wonbio-96c multi-sample frozen grinding machine (Shanghai Wanbai Biotechnology Co., Ltd., China), a Watson VS-2500MS digital display timing vortex mixer (Wuxi Watson Instrument Manufacturing Co., Ltd., China), an intelligent constant temperature tank ultrasonic extractor Scientz-5 TQL 4 (Ningbo Xinzhi Biotechnology Co., Ltd., China), a Centrifuge 5430 R refrigerated centrifuge (Eppendorf, China), the Vanquish Horizon UHPLC Liquid chromatography system (Thermo Fisher Scientific, America), and a Q Exactive system mass spectrometer (Thermo Fisher Scientific, America).

### 2.3 Sample processing and testing

#### 2.3.1 Extraction of leaf metabolites

50 mg *Phlomoides rotata* leaves was added to a 2-mL centrifuge tube, and a 6-mm-diameter grinding bead was added. 400 μL of extraction solution (methanol: water = 4:1 (v: v)) containing 0.02 mg/mL of internal standard (L-2-chlorophenylalanine) was used for metabolite extraction. Samples were ground by the Wonbio-96c (Shanghai Wanbo Biotechnology Co., Ltd.) frozen tissue grinder for 6 min (−10°C, 50 Hz), followed by low-temperature ultrasonic extraction for 30 min (5°C, 40 kHz). The samples were left at −20°C for 30 min and centrifuged for 15 min (4°C, 13,000 g), and the supernatant was transferred to the injection vial for LC-MS/MS analysis.

#### 2.3.2 LC-M S/MS analysis

As a part of the system conditioning and quality control process, a pooled quality control sample (QC) was prepared by mixing equal volumes of all samples. The QC samples were disposed and tested in the same manner as the analytic samples. It helped to represent the whole sample set, which would be injected at regular intervals (every 5–15 samples) in order to monitor the stability of the analysis.

The chromatographic conditions are as follows: the column is Waters ACQUITY UPLC BEH C18 column (100 mm 2. 1 mm, 1. 7 µm), mobile phase A is water (containing 0. 1% formic acid), mobile phase B is acetonitrile/isopropyl alcohol (1/1) (containing 0. 1% formic acid), the sample injection is 10 μl, the column temperature is 40°C, and the flow rate is 0. 40 ml/min; the elution gradient is shown in **Table 2**. The UPLC system was coupled to a Thermo UHPLC-Q Exactive Mass Spectrometer equipped with an electrospray ionization (ESI) source operating in positive mode and negative mode. The optimal conditions were set as follows: source temperature at 400°C; sheath gas flow rate at 40 arbs; aux gas flow rate at 10 arb; ion-spray voltage floating (ISVF) at −2,800 V in negative mode and 3,500 V in positive mode; normalized collision energy, 20–40–60 V rolling for MS/MS. Full MS resolution was 70,000, and MS/MS resolution was 17,500. Data acquisition was performed with the data-dependent acquisition (DDA) mode. The detection was carried out over a mass range of 70–1,050 m/z. The MS ionization mode is electric spray ionization, the S-Lens voltage is 50 V, and the full-scan resolution is 60,000 (FWHM).

**Table 2:** Mobile phase elution gradient.

Time (min)	Flow rate (ml/min)	A (%)	B (%)
0	0.4	95	5
3	0.4	80	20
9	0.4	5	95
13	0.4	5	95
13. 1	0.4	95	5
16	0.4	95	5

### 2.4 Data processing

The LC-MS raw data were converted into the common format by Progenesis QI software (Waters, Milford, USA) through baseline filtering, and a three-dimensional data matrix in CSV format was exported. The information in this three-dimensional matrix included sample information, metabolite name, and mass spectral response intensity. Internal standard peaks, as well as any known false positive peaks (including noise, column bleed, and derivatized reagent peaks), were removed from the data matrix, de-redundant, and peak pooled. At the same time, the metabolites were identified by searching databases, and the main databases were HMDB (http://www.hmdb.ca/), Metlin (https://metlin.scripps.edu/), and the self-compiled Major Bio-Database (MJDB) of Major bio-Biotechnology Co., Ltd. (Shanghai, China). Concretely, the ppm error is set to be less than 10 ppm. For metabolites with MS/MS confirmation, only those with an MS/MS fragment score higher than 30 are considered confirmed.

The data matrix obtained by searching databases was uploaded to the Majorbio Cloud platform (https://cloud.majorbio.com) for data analysis. Firstly, the data matrix was preprocessed, as follows: At least 80% of the metabolic features detected in any set of samples were retained. After filtering, the minimum value in the data matrix was selected to fill the missing value and each metabolic signature was normalized to the sum. To reduce the errors caused by sample preparation and instrument instability, the response intensities of the sample mass spectrometry peaks were normalized using the sum normalization method, to obtain the normalized data matrix. Meanwhile, the variables of QC samples with relative standard deviation (RSD) >30% were excluded and log10 logarithmized, to obtain the final data matrix for subsequent analysis.

Then, the R package “ropls” (Version 1.6.2) was used to perform principal component analysis (PCA) and partial squares discriminant analysis (PLS-DA), as well as seven-cycle interactive validation evaluating the stability of the model. The metabolites with VIP>1 and P<0.05 were determined as significantly different metabolites based on the variable importance in the projection (VIP) obtained by the PLS-DA model and the P-value generated by Student’s t test.

Differential metabolites among two groups were mapped into their biochemical pathways through metabolic enrichment and pathway analysis based on the KEGG database (http://www.genome.jp/kegg/). These metabolites could be classified according to the pathways they involved or the functions they performed. Enrichment analysis was used to analyze a group of metabolites in a function node whether appears or not. The principle was that the annotation analysis of a single metabolite develops into an annotation analysis of a group of metabolites. Python packages “SciPy. Stats” (https://docs.scipy.org/doc/scipy/) was used to perform enrichment analysis to obtain the most relevant biological pathways for experimental treatments.

## 3 Results and analysis

### 3.1 *Phlomoides rotata* leaf metabolites

The metabolites in the preprocessed data tables were annotated, and the results are presented below in **Table 3**.

**Table 3:** Annotation information of *Phlomoides rotata* leaves metabolites.

Ion mode	All peaks	Identified metabolites	Metabolites in library	Metabolites in KEGG
pos	21,641	1,596	1,128	518
neg	19,764	1,540	1,320	361

ion mode: The mass spectrometer detects the ion mode of the substance; there are mainly pos (positive ion mode) and neg (negative ion mode). All peaks: the number of mass spectrum peaks extracted by the software. Identified metabolites: By using the primary and secondary mass spectrometry data, search for the library (self-built library, METLIN, HMDB, etc.), the final number of metabolites identified. Metabolites in library: The number of metabolites annotated to public databases such as HMDB and lipid maps. Metabolites in KEGG: the number of metabolites annotated to the KEGG database.

**Figure 2:**
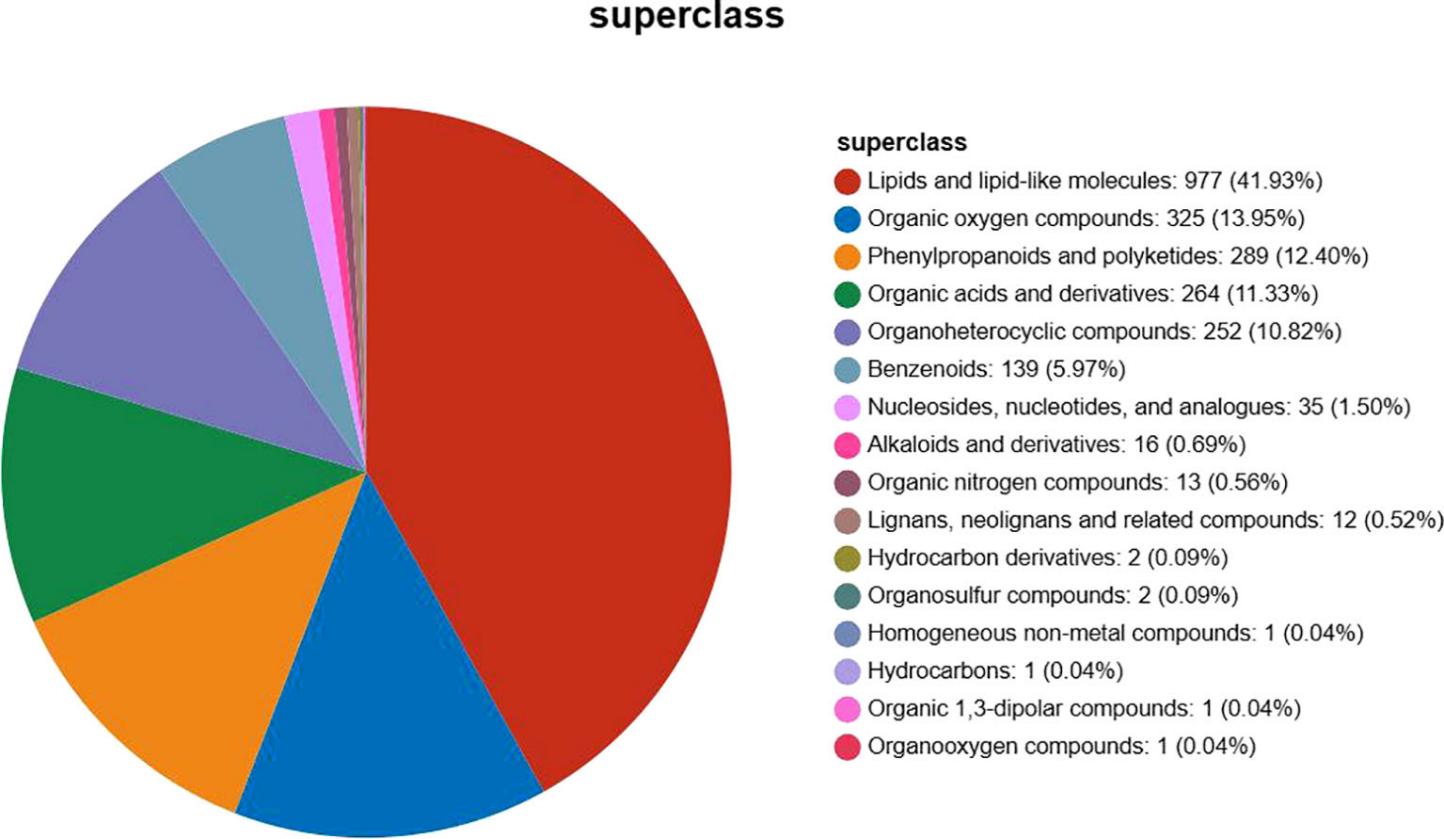
Classification statistics of HMDB-annotated metabolites in *Phlomoides rotata* leaves. The name of the selected HMDB tier and the percentage of metabolites accounted for are shown in descending order based on the number of metabolites. Different colors in each pie chart in the figure represent different HMDB classifications, and their area indicates the relative percentage of metabolites in that classification.

Using HMDB search, a total of 2,331 metabolites were detected, matching the metabolite secondary classification statistics, which were categorized into 16 species, as shown below in **Figure 2**. The summary table of metabolite information is shown in Attachment **Table 1**. HMDB-annotated metabolites in *Phlomoides rotata* leaves, lipid, and lipid-like molecules accounted for the largest proportion of 41.93%, followed by organic oxygen compounds, phenylpropanoids and polyketides, organic acids and derivatives, and organ heterocyclic compound, accounting for more than 10%.

Lipids play an important role in regulating normal cell physiology and function, and play an important physiological function in the process of plant response to abiotic stress (salt stress, drought stress, temperature stress, etc.) (Liu et al., 2021). Lipids have fewer anti-inflammatory properties, higher antioxidant effects, and immunomodulatory effects and can reduce the risk of cholestasis (Raman et al., 2017). GeoR et al. showed that the relationship between lipids and cancer is steadily increasing, involving the occurrence of cancer, proliferation, migration, and apoptosis (Guo et al., 2020).

Phenylpropanoids and polyketides account for 12.40% of the *Phlomoides rotata* leaf total annotated metabolites, and phenylpropane metabolites, especially lignin and flavonoids, have important regulatory functions on plant growth and development and plant–environment interactions (Dong and Lin, 2021). Phenylpropanoid is an important component of the cell wall and a pigment mediating plant–pollinator interactions (Agar and Cankaya, 2020). It can be used as antioxidants, UV barriers, and anticancer, antiviral, anti-inflammatory, wound healing, and antimicrobial agents (Korkina, 2007). Natural polyketide compounds and their derivatives have significant efficacy and promising clinical applications as antifungal agents (Wang et al., 2023). From the Rhodiola tibetica endophytic fungus, Alternaria (Alternaria sp. HJT-Y7) in the isolated polyketides has anti-inflammatory activity (Lu et al., 2023).

Organic acids and their derivatives account for 11.33%. Low molecular weight organic acids (LMWOA) are ubiquitous on the earth’s surface and are an important intermediate product in the metabolic pathway of organic matter, and they participate in the tricarboxylic acid cycle in life activities (Xiao and Wu, 2014). They have a wide role in plant stress resistance. Compared with other acids such as amino acids, OA is a more effective chelating agent and can also serve as a “carbon source “for microorganisms (Panchal et al., 2021).

In addition, the annotated metabolites are benzenoids, benzene compounds isolated from the fruiting body of camphor tree, showing effective inhibition of fMLP-induced superoxide production and having anti-inflammatory effects (Chen et al., 2007). For decades, nucleosides, nucleotides, and base analogs have been used clinically for the treatment of viral pathogens and tumors (Shelton et al., 2016). Galantamine as an Amaryllidaceae alkaloid has an important role in the treatment of Alzheimer’s disease (Keglevich et al., 2016). Alkaloids represent a potential novel natural antibiotic with a broad antimicrobial spectrum, rare adverse effects, and a low trend toward resistance (Yan et al., 2021). They have physiological and ecological functions in regulating plant growth, coping with environmental stresses, and preventing diseases and insect pests (Zhang et al., 2014). Organic nitrogen compounds can be used as a source of nitrogen nutrients for higher plants (Virtanen and Linkola, 1946). Lignans, neolignans, and related compounds have long been used in ethnic medicine and traditional medicine due to their antioxidant, antitumor, anti-inflammatory, antiviral and other biological activities (Teponno et al., 2016). Organosulfur compounds are an important class of therapeutic agents in medicinal chemistry due to their involvement in biosynthesis, metabolism, cellular function, and protection of cells from oxidative damage (Egbujor et al., 2022). Organosulfur compounds of garlic have a range of antibacterial properties, such as bactericidal, anti-biofilm, antitoxin, and anti-quorum sensing activity against a variety of bacteria (including multiple drug-resistant (MDR)strains) (Bhatwalkar et al., 2021). Diverse hydrocarbons can be used as important chemicals in the fields of food, fuel, pharmaceuticals, nutrition, and cosmetics (Xie et al., 2017). Crude oil-derived hydrocarbons are the world’s largest environmental pollutant (Ławniczak et al., 2020).

### 3.2 Correlation analysis

#### 3.2.1 PCA of the samples at different elevations

**Figure 3:**
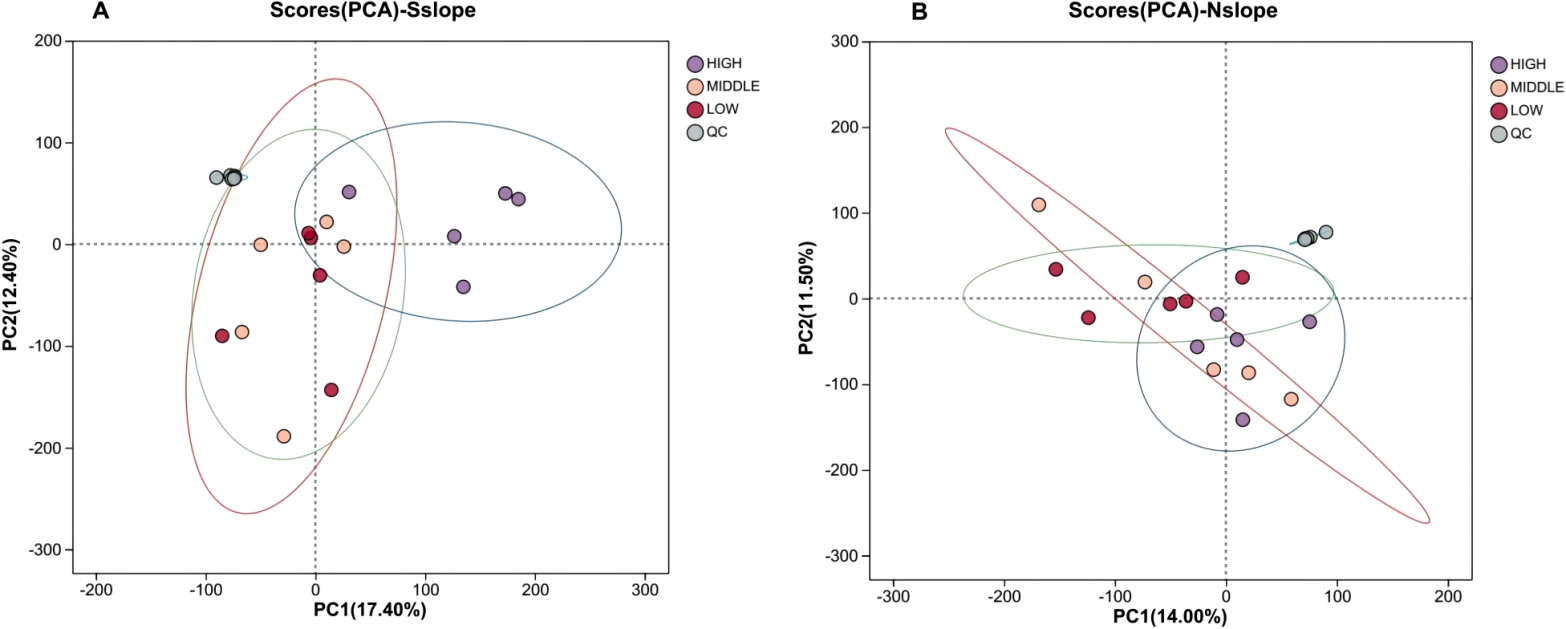
Scatter plot of PCA scores for different elevation samples. Panel **(A)** represents the PCA score scatter plot of south slope samples, and Panel **(B)** represents the PCA score scatter plot of north slope samples. HIGH is the high-altitude sample,MIDDLE is the middle- altitude sample, LOW is the low-altitude sample, and QC is the quality control sample.

A series of multivariate pattern recognition analyses were performed on the data to obtain a graph of the PCA scores of the samples. As shown in **Figure 3**, the QC samples showed aggregation and reliable data; the trend of discrete between samples within the group was not obvious and they were all within the confidence intervals, and the reproducibility between the samples within the group was relatively good. There are differences in the metabolite profiles of 15 samples from each of the three altitude gradients on the southern and northern slopes in the PC1 and PC2 directions, with a total contribution rate of 29.80% on the southern slope and 25.50% on the northern slope. The general distribution trend of the samples could be observed, but there was an obvious crossover phenomenon among the three groups of samples, so a more effective model was sought to explain the metabolic differences between the two groups of samples.

#### 3.2.2 The PLS-DA of the samples at different elevations

**Figure 4:**
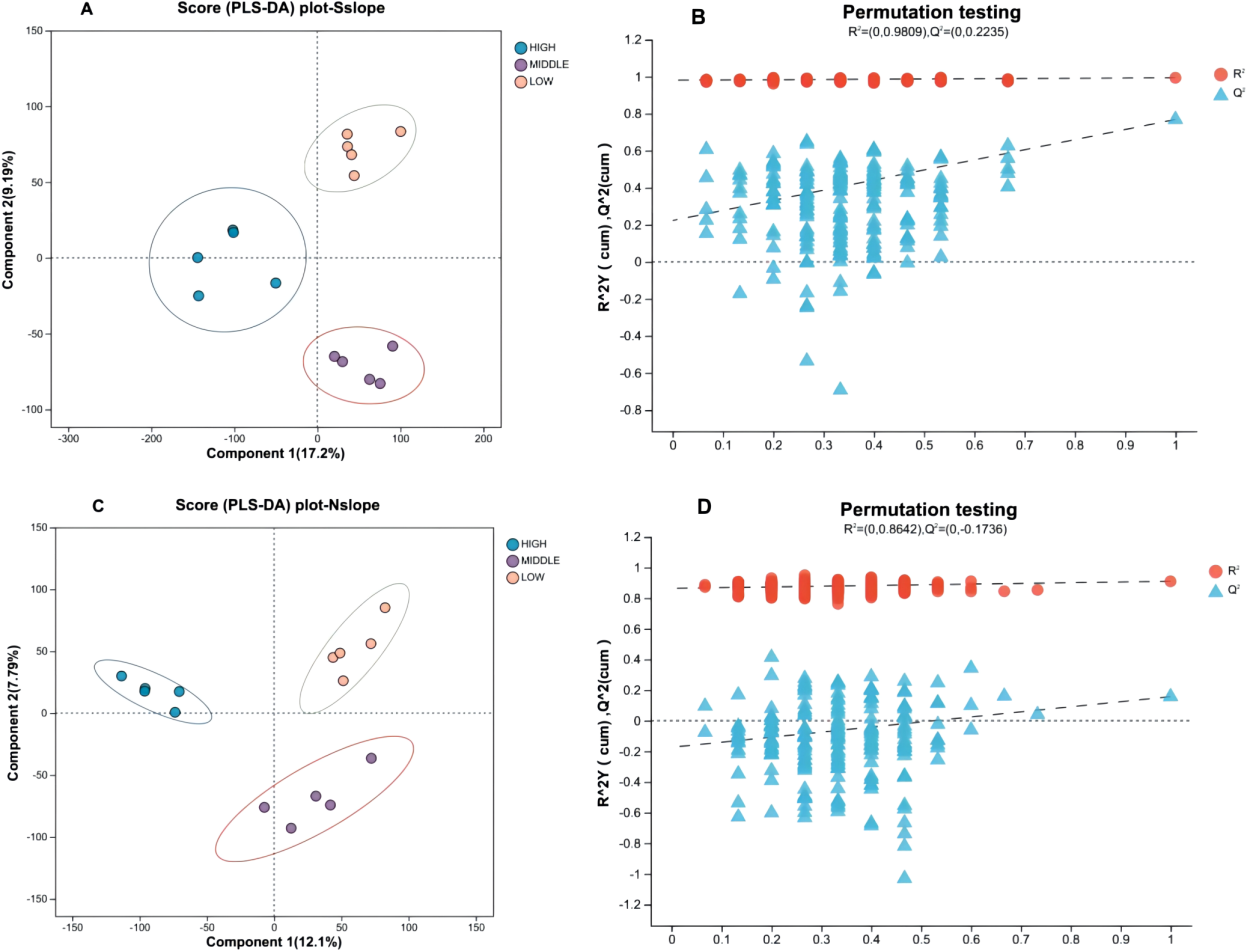
PLS-DA score plots with permutation test for different elevation samples. Panels **(A, B)** correspond to the PLS-DA score plot and permutation test plot of south slope samples, respectively, with panels **(C, D)** representing the same for north slope samples. The left graph Component 1 first principal component explanatory degree, Component 2 second principal component explanatory degree; the right graph horizontal coordinates indicate the replacement retention of the replacement test, vertical coordinates indicate the values of R^2^ (red dots) and Q2 (blue triangles) replacement test, and the two dashed lines indicate the regression lines of R^2^ and Q^2^, respectively.

To further distinguish between-group differences, the data were analyzed by supervised PLS-DA. According to **Figure 4**, each sample is effectively separated, and the degree of aggregation within each group is more aggregated than the PCA method. The PLS-DA model was tested 200 times and showed good explanatory power (South slope R2 = 0.9809, North slope R2 = 0.8642), and as the displacement retention decreases, the Q2 regression line shows an upward trend, indicating that the replacement test is passed, and the model has no overfitting phenomenon.

#### 3.2.3 PCA of samples with different slope directions

**Figure 5:**
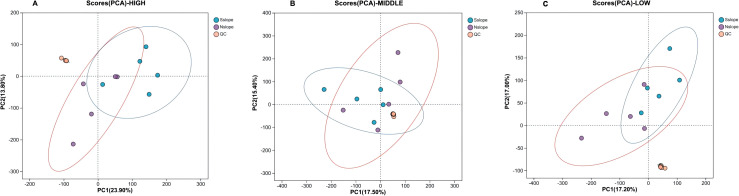
Scatter plot of PCA scores for samples with different slope directions. Panel **(A)** represents the PCA score scatter plot of high-altitude samples from different slope aspects. Panel **(B)** represents the PCA score scatter plot of medium-altitude samples from different slope aspects. Panel **(C)** represents the PCA score scatter plot of low-altitude samples from different slope aspects.

From **Figure 5**, we know that the samples from the north and south slopes in the high-, middle-, and low-altitude groupings differed in both the PC1 and PC2 directions, but there was an obvious crossover phenomenon between the samples, so we searched for a more effective model to reveal their metabolic differences.

#### 3.2.4 PLS-DA of samples with different slope directions

**Figure 6:**
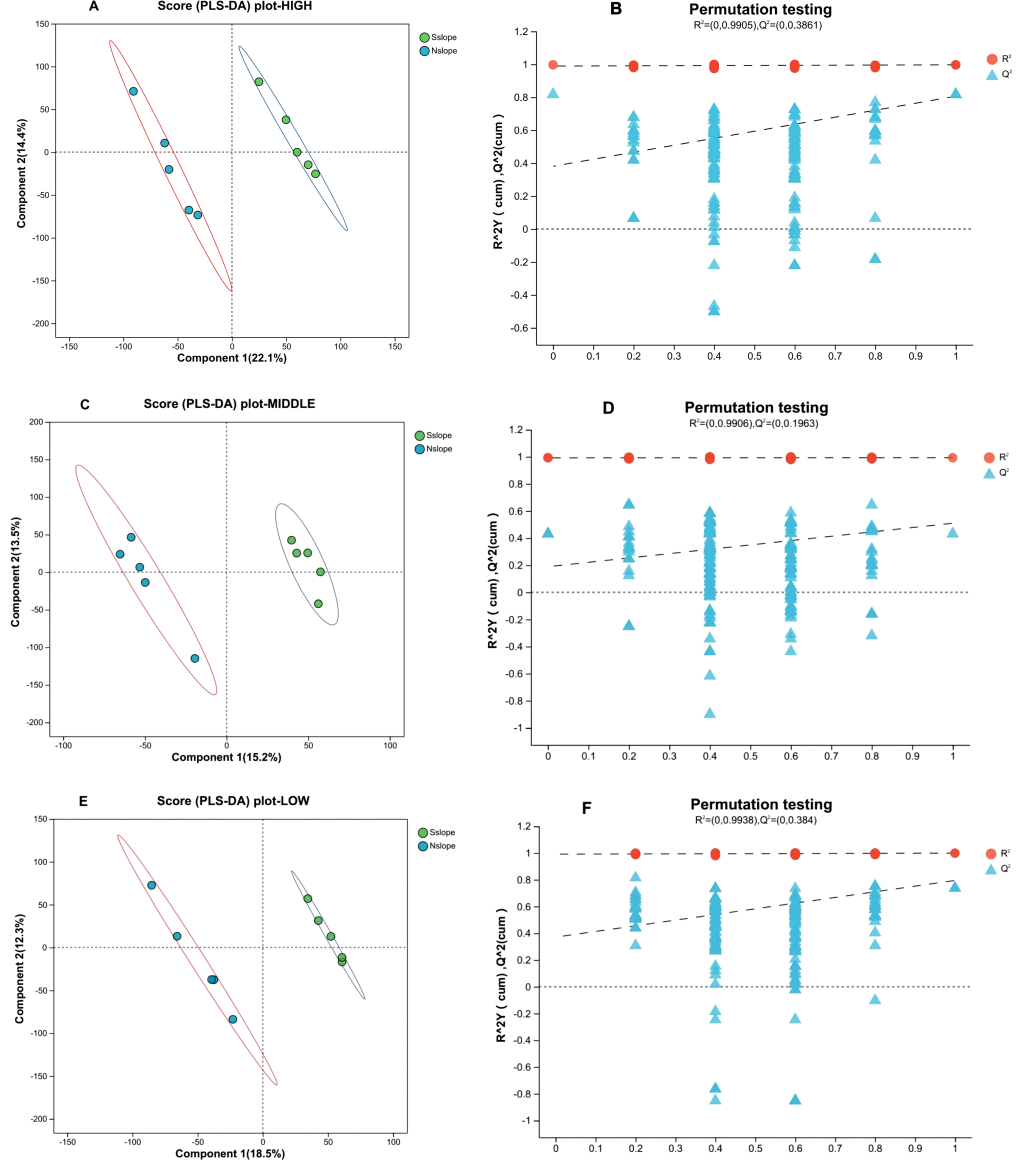
Plot of PLS-DA scores with permutation test for samples with different slope directions. Panels **(A, B)** represent the PLS-DA score plot and permutation test plot of high-altitude samples from different slope aspects, respectively. Panels **(C, D)** represent the PLS-DA score plot and permutation test plot of medium-altitude samples from different slope aspects, respectively. Panels **(E, F)** represent the PLS-DA score plot and permutation test plot of low-altitude samples from different slope aspects, respectively.

For the need to distinguish between groups, data were analyzed by the supervised PLS-DA method. **Figure 6** shows that the two samples of the north and southern slopes of the three-elevation gradient were effectively separated, and the degree of aggregation within each group of samples was also more aggregated. After 200 displacement tests, the model has good explanatory power (high altitude R2 = 0.9905, mid-elevation R2 = 0.9906, low elevation R2 = 0.9938), and as the displacement retention decreases, the Q2 regression line shows an upward trend, indicating that the replacement test is passed, and the model has no overfitting phenomenon.

### 3.3 The screening of significantly differential metabolites

#### 3.3.1 Screening of metabolites with significant differences in samples of different altitudes

A multigroup analysis of variance (ANOVA) was performed on each of the three elevation samples from the south and north slopes to compare the distribution of metabolites in the three sample groups for significant differences using the Kruskal–Wallis H test, and then a post-hoc test was performed on the metabolites that differed to screen for significantly different metabolites based on a P value<0. 05.

**Figure 7:**
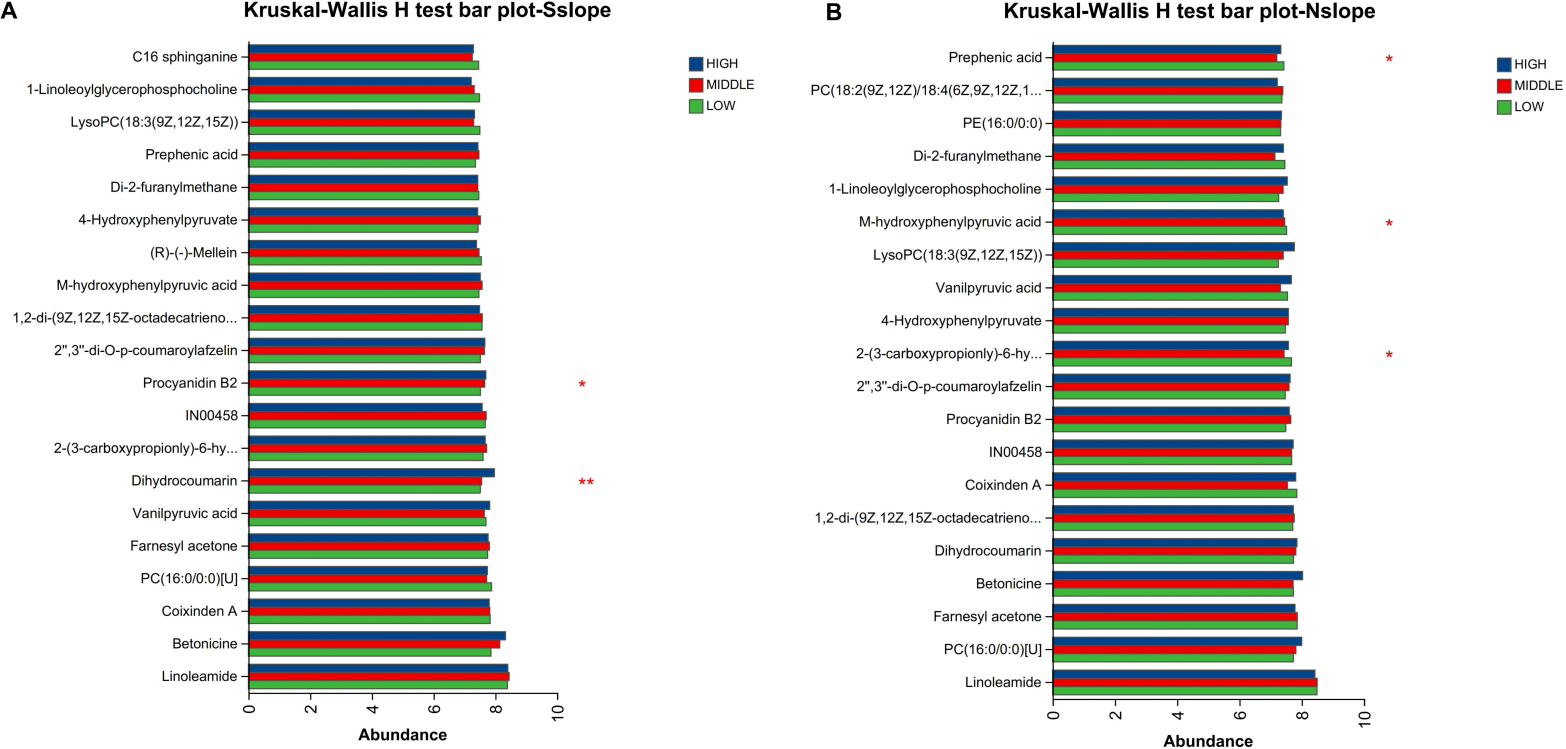
Comparison of differential metabolites of different elevations of *Phlomoides rotata* leaves on the north and south slopes bar charts. Panel **(A)** represents the differential metabolites of samples from different altitudes on the south slope. Panel **(B)** represents the differential metabolites of samples from different altitudes on the north slope. The Y-axis represents the metabolite name, the X-axis represents the average relative abundance of metabolites in different groups, and different colored columns indicate different groups; the far right is the P value, *0. 01<P ≤ 0. 05, **0. 001<P ≤ 0. 01.

**Figure 8:**
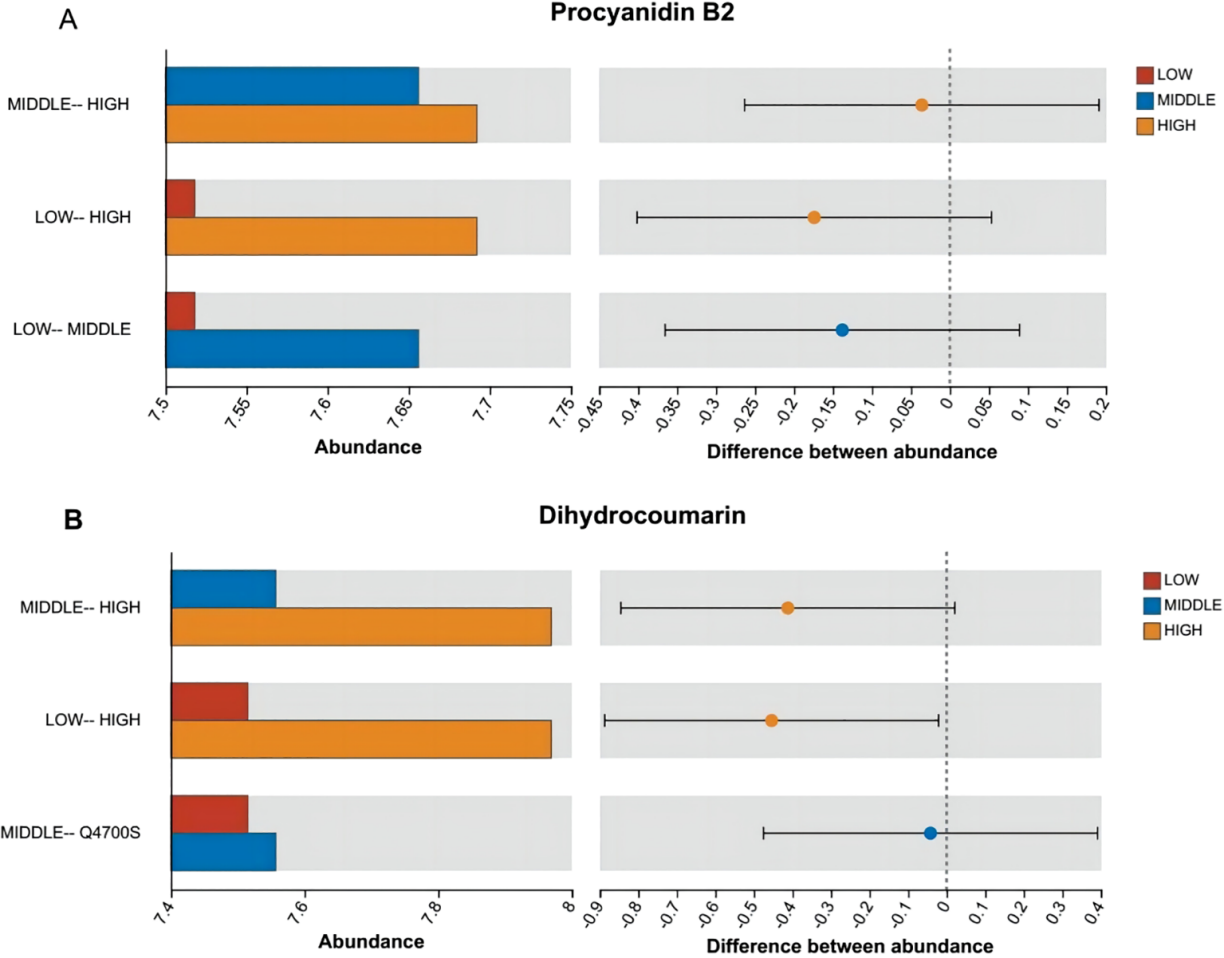
Histogram of the difference in the distribution of the abundance of two substances among multiple groups. Panel **(A)** represents the differential abundance distribution of Procyanidin B2 across different altitudes on the south slope. Panel **(B)** represents the differential abundance distribution of Dihydrocoumarin across different altitudes on the south slope. The X-axis of the left bar chart represents the average relative abundance of a metabolite in different groups; the ordinate represents the pairwise categories, and different colors indicate different groups. The right area is the set confidence interval. The value corresponding to the dots represents the difference in the average relative abundance of metabolites in the two groups. The dots show the color of the metabolite abundance, and the type I interval on the dots is the upper and lower limit of the difference.

**Figure 9:**
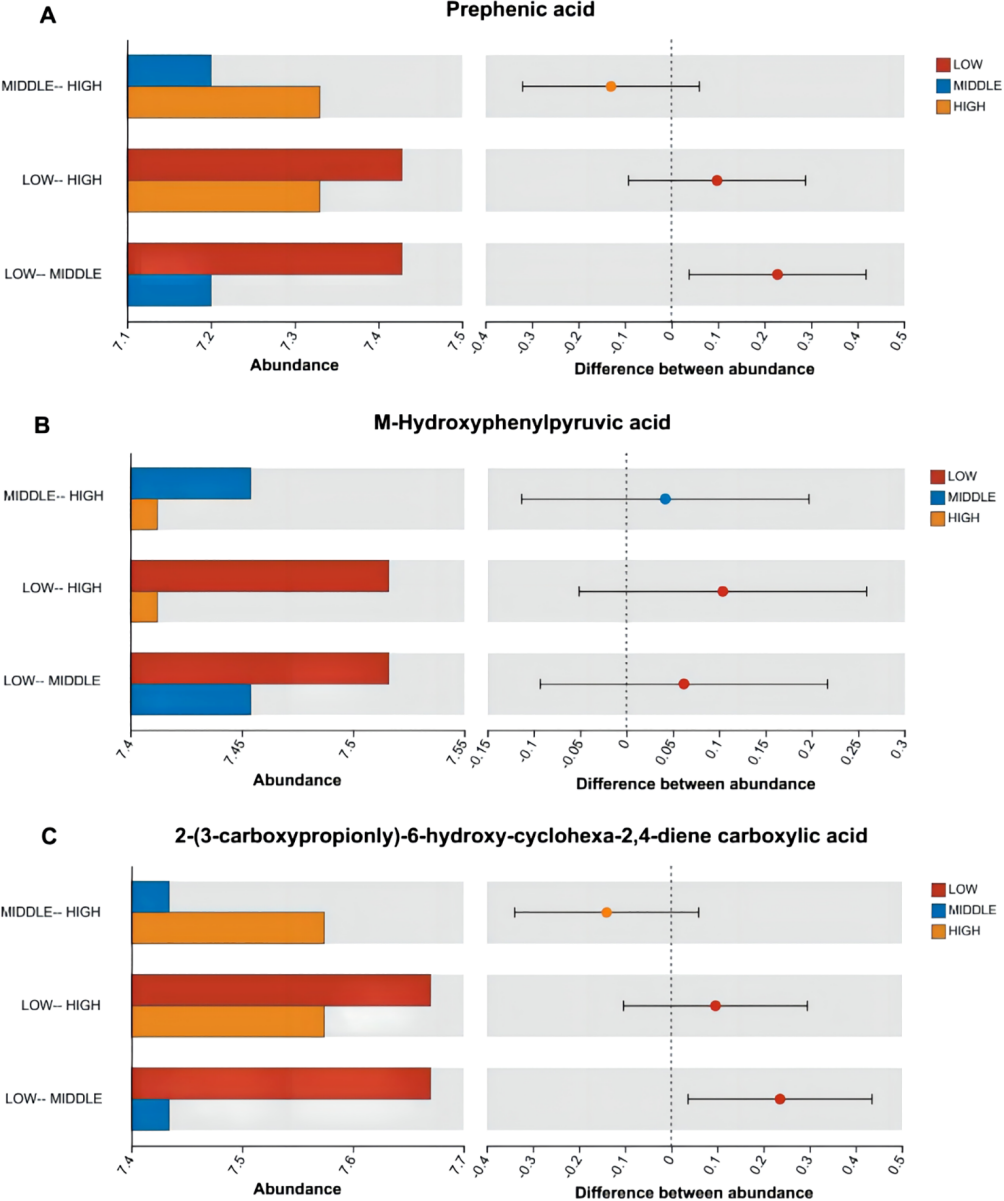
Histogram of differences in distribution of abundance of three substances among multiple groups. Panel **(A)** represents the differential abundance distribution of Prephenic acid across different altitudes on the north slope. Panel **(B)** represents the differential abundance distribution of M-Hydroxyphenylpyruvic acid across different altitudes on the north slope. Panel **(C)** represents the differential abundance distribution of 2-(3-Carboxypropionyl)-6-hydroxy-cyclohexa-2,4-diene carboxylic acid across different altitudes on the north slope.

According to **Figures 7**–**9**, two significantly different metabolites at different elevations on the south slope were procyanidin B2 and dihydrocoumarin, and three significantly different metabolites on the north slope were prephenic acid, M-hydroxyphenylpyruvic acid, and 2-(3-carboxypropionyl)-6-hydroxy-cyclohexa-2,4-diene carboxylic acid. Molecular masses and molecular formulas are shown in **Tables 4**, **5**.

**Table 4:** Differential metabolite statistics of different elevations of *Phlomoides rotata* leaves on the south slope.

NO.	Metabolite	KEGG pathway first category	m/z	Formula
1	Procyanidin B2	–	579.1479	C_30_H_26_O_12_
2	Dihydrocoumarin	–	166.0860	C_9_H_8_O_2_

① Procyanidin B2, with a relative molecular mass of 578.52, is a water-soluble pigment belonging to flavonoid compounds with anti-inflammatory and antioxidant properties (Baba et al., 2002; Tanaka et al., 2017). The average relative abundance was higher at high and middle elevations. As an important natural compound, flavonoids have various physiological activities such as antioxidation, anti-inflammatory, antiaging, anticancer, and antiviral and have great application potential in food, cosmetics, medicine, and other industries (Feng and Wang, 2021). The Cassia bark proanthocyanidin-B2 component has anti-saccharification potential, and food sources rich in proanthocyanidin such as proanthocyanidin-B2 can control AGE-mediated diabetic complications (Muthenna et al., 2013). Proanthocyanidin B2 attenuates FFA-induced hepatic steatosis by modulating the TFEB-mediated lysosomal pathway, and the redox status may represent promising new drugs for the prevention and treatment of non-alcoholic fatty liver disease (NAFLD) (Su et al., 2018). It can also have protective effects against CCl4-induced liver injury by enhancing the antioxidant defense potential, thereby inhibiting the inflammatory response and apoptosis in liver tissue (Yang et al., 2015).

② Dihydrocoumarin, with a relative molecular mass of 148.16, belongs to the 3,4-dihydrocoumarin compound, with irritant and acute toxicity (Program, 1993). Mean relative abundance was higher at higher elevations and lower at middle and lower elevations. Dihydrocoumarin can be developed as a new group-sensing inhibitor or antibiofilm agent to control food spoilage and may be investigated to improve food safety (Hou et al., 2017). It can be used as a biological herbicide to control the growth of rice transplanting fields (Yang et al., 2022). 3,4-Dihydrocoumarin causes foregut ulcers, hyperplasia, inflammation, and parathyroid hyperplasia and increases the severity of nephropathy in male rats (Program, 1993).

**Table 5:** Differential metabolite statistics of *Phlomoides rotata* leaves at different elevations on the North Slope.

No.	Metabolite	KEGG pathway first category	m/z	Formula
1	Prephenic acid	Metabolism	209.0439	C_10_H_10_O_6_
2	M-Hydroxyphenylpyruvic acid	–	163.0385	C_9_H_8_O_4_
3	2-(3-Carboxypropionyl)-6-hydroxy-cyclohexa-2,4-diene carboxylic acid	–	241.0700	C_11_H_12_O_6_

① Prephenic acid, with a relative molecular mass of 226.18, belongs to γ-keto acid and its derivative compounds and is an intermediate in the biosynthesis of aromatic compounds. The mean relative abundance is higher at lower elevations. High concentrations of prephenic acid were able to inhibit the inhibitory effect of NAD on E. coli tRNA (Dénes and Nagy, 1976).

② M-Hydroxyphenylpyruvic acid, with a relative molecular mass of 180.16, belongs to a phenylpyruvate derivative. The mean relative abundance is higher at lower elevations.

③ 2-(3-Carboxypropionyl)-6-hydroxy-cyclohexa-2,4-diene carboxylic acid, with a relative molecular mass of 240.21, belongs to an organic oxygen compound with a high average relative abundance at low altitude.

#### 3.3.2 Screening of metabolites with significant differences in samples from different slope directions

**Figure 10:**
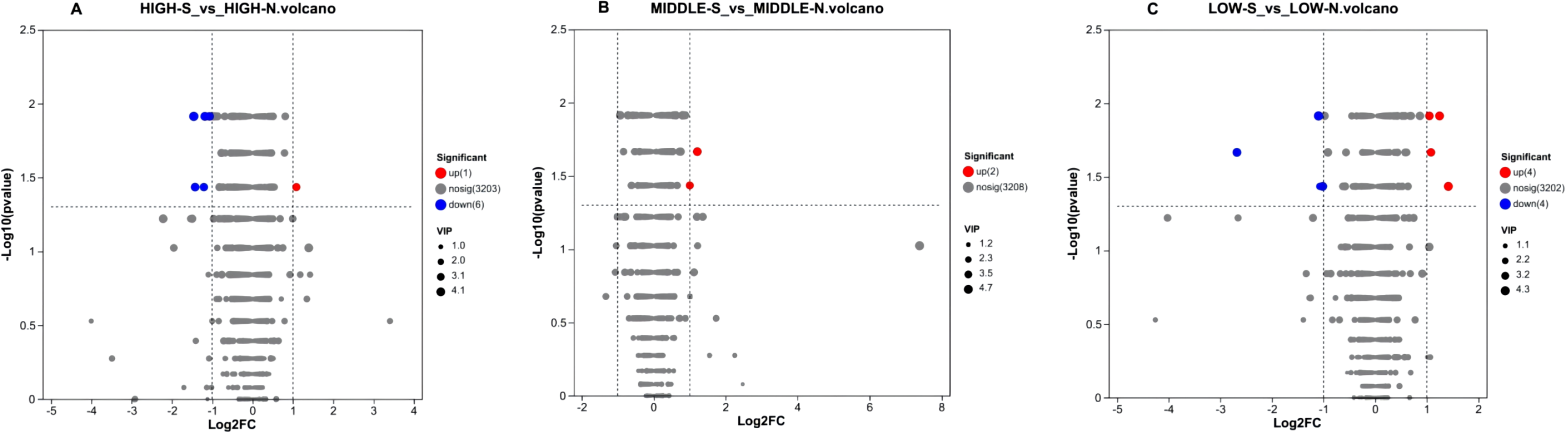
Volcano plot of metabolite differences between leaves of *Phlomoides rotata* at three altitudes north and south slopes. Panels **(A–C)** represent the volcano plots of differential secondary metabolites at high, medium, and low altitudes, respectively. The abscissa is the fold change value of the expression difference of the metabolite between the two groups, namely, log2FC, and the ordinate is the statistical test value of the difference in the expression difference of the metabolite, which is the-log10(P value). Each point represents a specific metabolite, and the size of the dot represents the VIP value. Red dots indicate significantly upregulated metabolites, blue dots are significantly downregulated metabolites, and gray dots are non-significantly different metabolites.

In this experiment, the variable projected importance (VIP) value >1 and P-value <0. 05 were used as the screening criteria to preliminarily screen out the differential metabolites among groups, and univariate statistical analysis was further used to screen out the metabolites with relative abundance difference multiplicity of more than 2-fold and to verify whether the differential metabolites were significant or not. Finally, we obtained volcano plots of the differences in secondary metabolites of *Phlomoides rotata* leaf samples from north and south slopes of the same altitude with P values <0. 05, VIP values >1 and fold of difference (FC)>2 (**Figure 10**).

Seven significantly differential metabolites were identified among HIGH-S vs. HIGH-N groups, including one upregulated and six downregulated (**Table 6**), two in MIDDLE-S vs. MIDDLE-N groups (**Table 7**), and eight among LOW-S vs. LOW-N groups, including four up- and four downregulated (**Table 8**).

**Table 6:** Statistics of metabolite differences between the leaves of *Phlomoides rotata* from north and south slopes at high altitude.

No.	Metabolite	Regulate	m/z	Formula
1	2, 3-Secoporrigenin	Down	441.2629	C_27_H_40_O_6_
2	Alpha-Micropteroxanthin B	Down	441.2992	C_27_H_40_O_2_
3	Perulactone	Down	539.2968	C_30_H_46_O_7_
4	Pubescenol	Down	473.2890	C_32_H_40_O_10_
5	Esculentoside E	Down	649.3570	C_35_H_54_O_11_
6	(x)-2-Heptanol glucoside	Up	301.1639	C_13_H_26_O_6_
7	Lysopa (0: 0/18: 0)	Down	483.2736	C_21_H_43_O_7_P

① 2, 3-Secoporrigenin, with a relative molecular mass of 460.60, belongs to the naphthofuran class of compounds.

② Alpha-Micropteroxanthin B, relative molecular mass of 396.61, belongs to steroidal compounds.

③ Perulactone, with a relative molecular mass of 518. 68, belongs to an ergosteroid steroid (Gottlieb et al., 1980), whose structure is characterized by bile acids or alcohols with three hydroxyl groups. The perulactone was positively correlated with theα-amylase inhibitory activity (Mahana et al., 2022).

④ Pubescenol, with a relative molecular mass of 584. 26, belongs to steroid lactones and their derivatives. Along with other substances, it was shown to be a moderate inhibitor of the growth of MCF-7, NCI-H460, and SF-268 human tumor cell lines (Valente et al., 2004).

⑤ Esculentoside E, with a relative molecular mass of 650.80, belongs to triterpenoid.

⑥ (x)-2-Heptanol glucoside, with a relative molecular mass of 278.34, belongs to the fatty acyl glycoside of monosaccharide and disaccharide.

⑦ Lysopa (0:0/18:0), with a relative molecular mass of 438.54, belongs to the glycerophospholipid compounds. It often exists in feces, so this substance may be a residual pollutant on the *Phlomoides rotata* leaves.

**Table 7:** Statistics on the differences in metabolites of *Phlomoides rotata* leaves at the north and south slopes of middle elevation.

NO.	Metabolite	Regulate	m/z	Formula
1	2-O-Alpha-D-galactopyranosyl-1-deoxynojirimycin	Up	689.2439	C_12_H_23_NO_9_
2	3-Alpha-hydroxyglycyrrhetinic acid	Up	469.3304	C_30_H_46_O_4_

① 2-O-Alpha-D-galactopyranosyl-1-deoxynojirimycin, relative molecular mass of 325.31, belongs to the adjacent glycocompound.

② 3Alpha-hydroxyglycyrrhetinic acid, with a relative molecular mass of 470.70, is a pentacyclic triterpenoid compound.

**Table 8:** Statistical on the differences in metabolites of *Phlomoides rotata* leaves at the north and south slopes of low altitude.

No.	Metabolite	Regulate	m/z	Formula
1	Xenognosin A	Down	237.0910	C_16_H_16_O_3_
2	Myrigalone A	Up	599.2691	C_18_H_20_O_4_
3	Alpha-Micropteroxanthin B	Down	441.2992	C_27_H_40_O_2_
4	Naringin 6″-rhamnoside	Up	771.2319	C_33_H_42_O_18_
5	2-Hydroxyiminostilbene	Down	273.1014	C_14_H_11_NO
6	Merodesmosine	Up	437.2160	C_18_H_34_N_4_O_6_
7	Crotaline	Up	326.1590	C_16_H_23_NO_6_
8	Lucyoside K	Down	677.3861	C_36_H_56_O_9_

① Xenognosin A, with a relative molecular mass of 256.30, belongs to phenolic compounds. Xenognosin regulates seed germination, host attachment organs, and several later stages of host–parasite fusion (Keyes et al., 2000).

② Myrigalone A, with a relative molecular mass of 300.35, belongs to monoterpene compounds. Milidone A photoinduces the oxides of terpenes, which in turn protects them from photolysis (Khaled et al., 2019). It can reduce the molecular mechanism of ethylene biosynthesis and inhibit seed germ growth (Heslop-Harrison et al., 2024). It plays its phytotoxic activity through a variety of molecular mechanisms dependent on and independent of accessory tin (Nakabayashi et al., 2022).

③ Alpha-Micropteroxanthin B, with a relative molecular mass of 396.61, belongs to steroidal compounds.

④ Naringin 6″-rhamnoside has a relative molecular mass of 726.68. They belong to the class of organic compounds called flavonoids-7-O-glycosides, which are phenolic compounds containing flavonoid moieties.

⑤ 2-Hydroxyiminostilbene, with a relative molecular mass of 209.24, belongs to the organic heterocyclic compound. 2-Hydroxyiminostyrene, a metabolite of carbamazepine, can activate the inflammasome, which may contribute to hypersensitivity in some patients (Kato et al., 2019). Following direct oxidation of 2-hydroxycarbamazepine to CBZ-IQ by cytochrome P450(P450s) followed by NADPH-mediated reduction to 2-hydroxyiminostyrene, these intermediates may play a role in the etiology of the specific heterogeneous toxicity of carbamazepine (Pearce et al., 2005).

⑥ Merodesmosine, with a relative molecular mass of 402.49, is an α-amino acid belonging to carboxylic acid and its derivatives. Merodesmosine is another crosslinker of elastin (Oishi et al., 2023).

⑦ Crotaline, with a relative molecular mass of 325.36, is an alkaloid with acute toxicity and suspected to be carcinogenic. For induction of the pulmonary hypertension model in rodents, crotaline has potent antitumor activity and is a natural ligand with dose-dependent cytotoxicity (Kusuma et al., 2014).

⑧ Lucyoside K, with a relative molecular mass of 632.82, is a triterpenoid saponin.

### 3.4 Pathway analysis and enrichment analysis of differential metabolites

Pathway enrichment analysis of differential metabolites helps to understand and analyze the mechanisms of changes in metabolic pathways. Metabolic pathways were detected by KEGG PATHWAY data annotation, and pathway enrichment analysis was performed with the following results.

#### 3.4.1 Pathway analysis of *Phlomoides rotata* leaf different metabolites in different elevations

**Figure 11:**
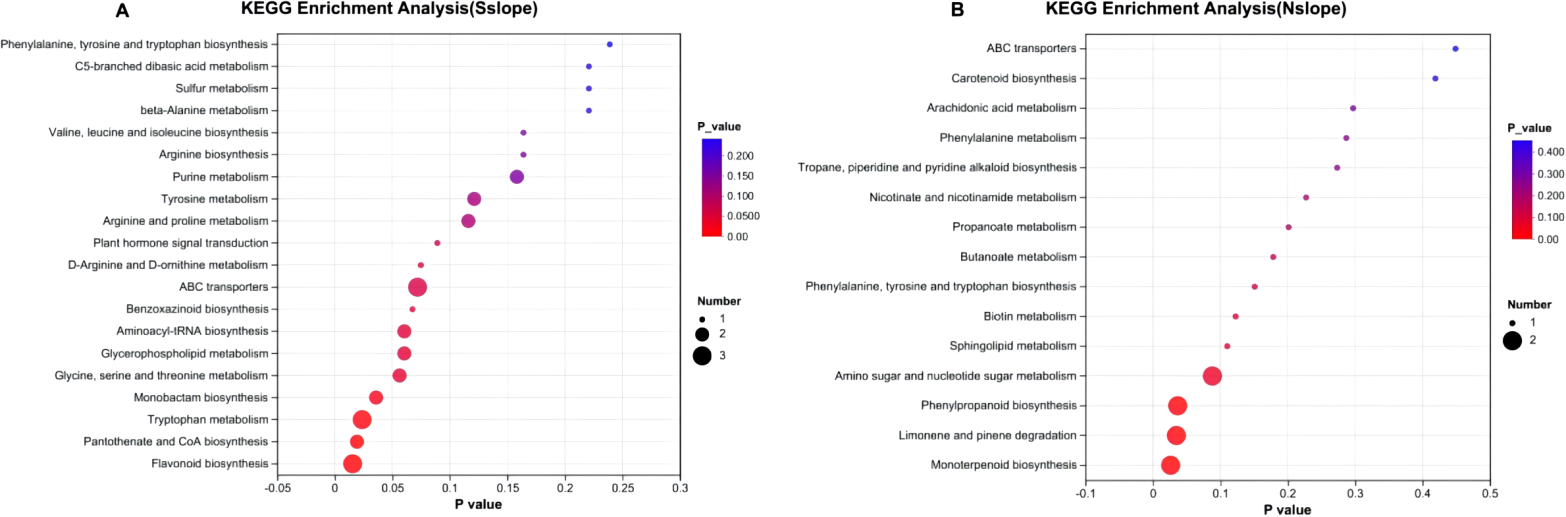
KEGG enrichment of metabolites of *Phlomoides rotata* leaves at different elevations of north and south slopes. Panel **(A)** represents the KEGG enrichment results of metabolites in samples from different altitudes on the south slope. Panel **(B)** the KEGG enrichment results of metabolites in samples from different altitudes on the north slope. The abscissa is the enrichment significance P-value; the ordinate is the KEGG pathway. The size of the bubble represents how much the pathway is enriched to the compound in the metabolic set.

The differential metabolites at different elevations were mainly distributed in seven pathways. As shown in **Figure 11**, there were four obvious differential metabolic pathways in the three elevation gradients in the south slope, which were flavonoid biosynthesis, pantothenate and CoA biosynthesis, tryptophan metabolism, and monobactam biosynthesis. The significantly different metabolic pathways on the north slope were three, monobactam biosynthesis, limonene and pinene degradation, and phenylpropanoid biosynthesis.

The most significant differential metabolic pathway on the southern slope is flavonoid biosynthesis: flavonoid compounds have many important biological functions, are the main source of plant pigments, and have potential beneficial effects on human health. As antibacterial drugs, they play an important role in the interaction and defense response between plants and microorganisms (Kusuma et al., 2014). It is found that low-temperature, moderate water drought favored the production and accumulation of plant flavonoids more (Zhao et al., 2021). Appropriate UV-B radiation as a promoter is important for the accumulation of flavonoid compounds, and high doses would inhibit their synthesis (Zhou et al., 2016). The most significant differential metabolic pathway on the North Slope was monoterpene biosynthesis, and increased environmental temperature and moderate drought could promote the production of monoterpenoids (Llusià and Peñuelas, 1998; Pandey and Agrawal, 2020). The UV also increases its production (Llusià and Peñuelas, 1998). The above research conclusions are consistent with our findings that *Phlomoides rotata*’s growth site undergoes changes in light, temperature, and humidity with altitude gradients, resulting in significant differences in various metabolic pathways.

#### 3.4.2 Pathway analysis of *Phlomoides rotata* leaves’ different metabolites in different slope directions

**Figure 12:**
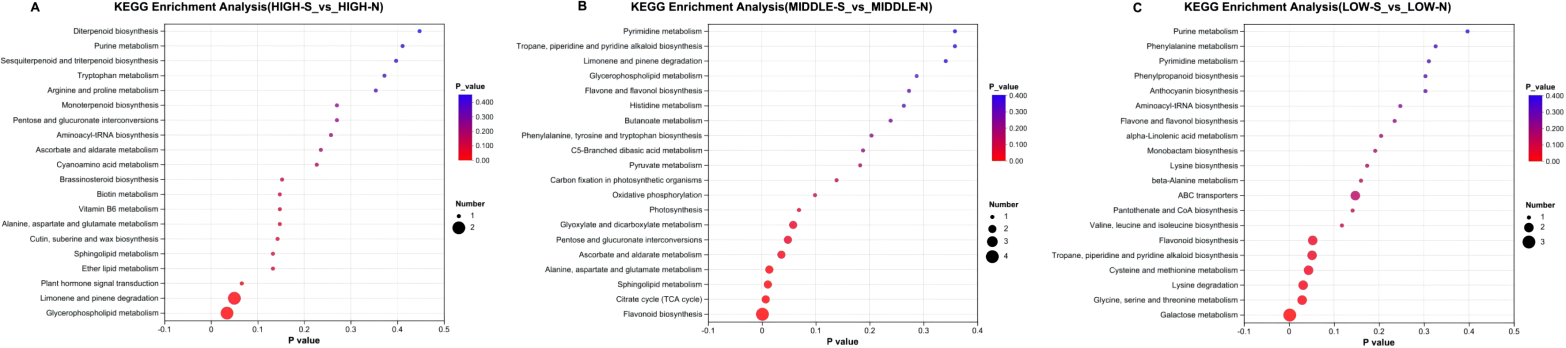
KEGG enrichment of metabolites of *Phlomoides rotata* leaves at the same elevation on the north and south slopes. Panel **(A)** represents the KEGG enrichment results of metabolites in samples from high altitudes on both the south and north slopes. Panel **(B)** represents the KEGG enrichment results of metabolites in samples from medium altitudes on both the south and north slopes. Panel **(C)** represents the KEGG enrichment results of metabolites in samples from low altitudes on both the south and north slopes.

The differential metabolites of different slopes were mainly distributed in the 11 pathways, as shown in **Figure 12**. There is only one apparent differential metabolic pathway of HIGH-S vs. HIGH-N, for glycerophospholipid metabolism. There are six distinct differential metabolic pathways of MIDDLE-S vs. MIDDLE-N: flavonoid biosynthesis; citrate cycle (TCA cycle); sphingolipid metabolism; alanine, aspartate, and glutamate metabolism; ascorbate and aldarate metabolism; and pentose and glucuronate interconversions. Four of LOW-S vs. LOW-N had distinct differential metabolic pathways: galactose metabolism; glycine, serine, and threonine metabolism; lysine degradation; and cysteine and methionine metabolism.

## 4 Discussion

At the level of traditional Tibetan medicine, it is believed that there are differences in the medicinal effects between artificially cultivated and wild plants of Angelica sinensis, which leads to serious overharvesting and overdigging of wild plants of Angelica sinensis, and the economic benefits of artificial cultivation cannot be realized, while also affecting the protection of wild plant resources. Zhao Xiaoyan systematically analyzed and evaluated the differences in characteristic components between wild Morchella and artificially cultivated Morchella using UPLC-Q-TOF-MS non-targeted screening technology. The results showed that hydroxamic acid-structured cyclic peptides and lipids were the main foreign substances, especially the hydroxamic acid-structured cyclic peptide compound MP-2, which was only detected in wild Morchella and had a high content. It can be used as a genuine biomarker to distinguish between wild collected and artificially cultivated Morchella (Zhao, 2022). This result indirectly reflects the possibility of differences between artificially cultivated and wild plants of *Phlomoides rotata*. Wang Junjie used UPLC-MS/MS-based metabolomics technology to sequence Aconitum pendulum Busch samples from six different habitat conditions. It was found that the main components of metabolites were diterpenoid alkaloids, and there were geographical differences in the types and contents of alkaloids in samples from different habitats (Wang, 2022). This study explored the differences in secondary metabolites of *Phlomoides rotata* leaves at three different altitude gradients and two north–south slope orientations using LC-MS from a non-targeted metabolomics perspective. The results showed that a total of 2,331 metabolites were detected using HMDB retrieval, with five differentially expressed metabolites identified at different altitudes and 17 identified at different slope orientations. Wang Luhao conducted metabolomic analysis on the leaves and roots of *Phlomoides rotata* from different regions of Qinghai Province and detected 589 metabolites, including 69 amino acids and their derivatives, 66 sugars, alcohols, and terpenes, 63 flavonoids and organic acids, and 62 alkaloids. There are differences in the quantity and composition of metabolites between the two, which may be caused by different habitats and soil conditions. Soil nutrients play a crucial role in the growth and development of plants, influencing the biosynthesis and accumulation of secondary metabolites. There are differences in the physicochemical properties of *Phlomoides rotata* soils in different regions, which may be one of the important reasons for the differences in the content of iridoid compounds in *Phlomoides rotata*. In future research, we can conduct physical and chemical property testing on the soil at each sampling point of *Phlomoides rotata*, in order to explore how the soil affects the composition and accumulation of *Phlomoides rotata* metabolites and the specific mechanism of action (Wang, 2024). KEGG enrichment analysis shows the southern difference metabolites of the most significant enrichment pathway for flavonoid biosynthesis; the northern difference metabolites of the most significant enrichment pathway for monoterpene biosynthesis; and the high-, medium-, and low-altitude difference metabolites of the most significantly enriched pathway for glycerophospholipid metabolism, flavonoid biosynthesis, and galactose metabolism. Li Zhuxia et al. performed metabolomic sequencing on *Phlomoides rotata* leaves from four different habitats (3,540–4,270 m) in Henan County, Guoluo County, Yushu County, and Chengduo County, Qinghai Province, and evaluated the compositional characteristics of *Phlomoides rotata* flavonoid metabolites in the four habitats. The enrichment of KEGG indicates the relationship between the biosynthesis of most flavonoid metabolites and phenylpropanoid, flavonoids, and secondary metabolites. A total of 59 metabolites were identified as flavonoids, of which nine showed significant differences. This is consistent with the results of metabolomics analysis conducted by Li Zhijun et al. on different tissue parts of *Phlomoides rotata*, which found that the aboveground part of *Phlomoides rotata* generally contains flavonoids. Flavonoids are important plant secondary metabolites that enhance plant stress resistance and protect normal plant growth by participating in plant resistance responses to certain biotic and abiotic stresses (Li et al., 2024). Based on previous research findings, the metabolic pathways enriched in secondary metabolites are responsive to the environment in which they are located. In the larger environment formed by altitude and slope orientation, multiple environmental factors such as light, temperature, and humidity have an impact on their secondary metabolites. Through comparison of environmental factors, we found that the formation of flavonoids may be closely related to slope orientation. In summary, this study systematically revealed the metabolic differences in *Phlomoides rotata* leaves at different altitudes and slopes using non targeted metabolomics. A total of 2,331 metabolites were detected, divided into 16 categories, mainly lipids and lipid-like molecules (41.93%), organic oxygen compounds (13.95%), etc. *Phlomoides rotata* has different metabolic characteristics at different altitude gradients and slope orientations, and a total of 27 differential metabolites were screened. Among them, five differential metabolites, including proanthocyanidin B2, dihydrocoumarin, prebenzoic acid, and 4-hydroxyphenylpyruvic acid, were screened for three altitude gradients. A total of 17 differential metabolites, including 2,3-seoporrigenin, 2-O-alpha-D-galactopyranosyl-1-deoxynojirimycin, and xenogenic glycosaminoglycan A, were screened for two north–south slope orientations. *Phlomoides rotata* differential metabolites were enriched in 18 metabolic pathways, including flavonoid biosynthesis, pantothenic acid salt biosynthesis, and cocoa biosynthesis, as well as glycerophospholipid metabolism and galactose metabolism. The metabolite composition of *Phlomoides rotata* leaves is very rich, especially with a high content of flavonoids. We can extract and enrich active flavonoids from *Phlomoides rotata* for the development of biopesticides, overcoming the disadvantages of traditional chemical pesticides such as high toxicity, high residue, and susceptibility to drug resistance. By using active flavonoids as the lead and chemical structure modification and activity evaluation, more active flavonoid derivatives can be screened for the development of new green pesticides with stronger efficacy. At the same time, *Phlomoides rotata* has different metabolites under different environmental conditions, resulting in different metabolic pathways in its body. These differences may be related to its actual pharmacological effects. In future research, attention can be paid to the differences in metabolites between artificially planted *Phlomoides rotata* and wild plants, and appropriate planting conditions can be selected based on the differences, in order to reduce the differences in pharmacological effects between artificially planted *Phlomoides rotata* and wild plants. Moreover, attention can be paid to the efficacy of different metabolites under different environmental conditions, and *Phlomoides rotata* plants can be selectively cultivated in corresponding environments to enrich their medicinal products and enhance their efficacy.

## Data Availability

The original contributions presented in the study are included in the article/supplementary material. Further inquiries can be directed to the corresponding author.
